# Dysregulation of lncRNAs in NK cells from breast cancer patients: implications for NK cell functions

**DOI:** 10.1007/s00251-025-01383-x

**Published:** 2025-08-09

**Authors:** Mona Rady, Eman Mohamed, Ola Khorshid, Khaled Abou-Aisha

**Affiliations:** 1 Microbiology, Immunology and Biotechnology Department, Faculty of Pharmacy and Biotechnology, German University in Cairo (GUC), Cairo, Egypt; 2 Faculty of Biotechnology, German International University (GIU), New Administrative Capital, Cairo, Egypt; 3 Medical Oncology Department, National Cancer Institute (NCI), Cairo, Egypt

**Keywords:** LncRNAs, NK cells, Breast cancer, Epigenetic regulation of gene expression, Polycomb repressive complex 2 (PRC2), X chromosome inactivation (XCI)

## Abstract

Natural killer (NK) cells, a key component of the innate immune system, play a crucial role in detecting and eliminating cancer cells, contributing to cancer immune surveillance. Increasing evidence suggests that NK cell functions are regulated by epigenetic mechanisms, including the influence of long non-coding RNAs (lncRNAs). These lncRNAs, transcripts longer than 200 nucleotides, are emerging as important regulators of gene expression. In this study, we investigated the expression of 84 lncRNAs in NK cells isolated from the peripheral blood of patients with invasive breast cancer. Using lncRNA PCR array profiling, we identified 26 differentially expressed lncRNAs in circulating NK cells isolated from peripheral blood of breast cancer patients, with 10 genes showing significant downregulation and 16 genes showing significant upregulation. Gene ontology (GO) and functional enrichment analysis revealed that among the ten downregulated lncRNAs, four lncRNAs have deposited gene ontology terms and known biological functions. These lncRNAs are *PTENP1-AS* (fold change 0.27, *P* = 0.0206), *TSIX* (fold change 0.05, *P* = 0.0037), *XIST* (fold change 0.42, *P* = 0.007), and *CCAT1* (fold change 0.09, *P* = 0.0191). On the other hand, among the 16 upregulated lncRNAs, five lncRNAs have deposited gene ontology terms and known biological functions. These lncRNAs are *GNAS-AS1* (fold change 5.10, *P* = 0.0104), *MEG3* (fold change 46.85, *P* = 0.0138), *CDKN2B-AS1* (fold change 81.80, *P* = 0.0087), *HOTAIR* (fold change 6.86, *P* = 0.0042), and *AIRN* (fold change 7.97, *P* = 0.0379). Functional enrichment analysis indicated that the downregulated lncRNAs were mainly involved in biological processes such as chromatin organization, epigenetic regulation of gene expression, and dosage compensation via X chromosome inactivation, while the upregulated lncRNAs were linked to epigenetic regulation, genomic imprinting, and chromatin remodeling. These enrichments were identified using Bonferroni correction with an adjusted *P*-value < 0.05. Given the biological functions of the dysregulated lncRNAs in NK cells from breast cancer patients, this has the potential to significantly impact the antitumor functionality of NK cells, possibly contributing to the impaired immune surveillance and tumor control commonly observed in breast cancer patients. Understanding the dysregulation of lncRNAs in NK cells may provide critical insights into the mechanisms underlying impaired NK cell function in breast cancer, offering promising approaches for developing immunotherapies aiming at restoring NK cell activity in cancer patients.

**Supplementary Information:**

The online version contains supplementary material available at 10.1007/s00251-025-01383-x.

## Introduction

While previously considered “junk” or the “black matter” of the genome (Slack and Chinnaiyan 2019), non-protein coding regions of the genome have several important functions and regulatory roles. Long non-coding RNAs (lncRNAs) represent a novel class of non-coding RNA transcripts longer than 200 nucleotides that perform molecular functions that are distinct from encoding proteins (Atianand et al. 2017). Although the human genome contains tens of thousands to hundreds of thousands of lncRNAs, almost nothing is known about 99% of these RNA transcripts (Mattick et al. 2023). Recent studies showed that lncRNAs are implicated in many distinct cellular processes including—but not limited to—chromatin remodeling, epigenetic silencing, and regulation of gene transcription, mRNA stability, RNA splicing, translation, and protein transport and trafficking (Chen et al. 2017). The sequence and structure of lncRNA determine the type of molecule it will interact with: DNA, RNA, or protein. Advances in techniques such as next generation sequencing, bioinformatic analysis of cDNA libraries, and tiling arrays revealed that lncRNAs expression is (1) pervasive, (2) relatively lower than protein coding transcripts, and (3) strikingly cell-type-specific or tissue-specific (Heward and Lindsay 2014).

LncRNAs are expressed in a variety of immune cells and in different immunological contexts with, however, unknown functional relevance. Expression profiles, however, revealed high immune cell-type specificity (Hrdlickova et al. 2014). Very few studies, however, addressed the functions lncRNAs in NK cell. Moreover, there are sparse studies studying lncRNA alterations effects in the immune systems of cancer patients. Only recently, Fang et. al (2019) studied the role of the lncRNA *GAS5* in regulating NK cell functions in liver cancer patients. Expression analysis revealed that *GAS5* is downregulated in NK cells isolated from liver cancer patients (Fang et al. 2019). Moreover upon IL2 stimulation of NK-92 cells or human primary NK cells, *GAS5* expression was upregulated, suggesting a role of *GAS5* in regulating NK cell functions. Indeed, the knockdown of *GAS5* in primary human NK cells reduced NK cell cytotoxicity, degranulation, as well as IFN-γ production (Fang et al. 2019). Similarly Stein et. al. (2019) showed that the lncRNA *IFNG-AS1* is induced upon NK cell activation and that forced over expression of *IFNG-AS1* increases IFN-γ production by NK cells (Stein et al. 2019). Zhang et. al. (2016) identified NK cell-specific lncRNAs and *lnc-CD56* as a novel lncRNA regulating the expression of CD56 in NK cells (Zhang et al. 2016). Analysis of KIRs genes revealed the presence of a promotor in intron 2 that produces a spliced antisense lncRNA transcript (Wright et al. 2013). Overexpression of this KIR antisense lncRNA resulted in decreased expression of KIR proteins (Wright et al. 2013). Several cancer-associated lncRNAs are significantly overexpressed in natural killer/T-cell lymphoma (Baytak et al. 2017). In CD8 + T cells, a lincRNA termed *TMEVPG1* is located within the cluster of cytokine genes that controlled the response to Theiler’s virus infection (Vigneau et al. 2003). Another lncRNA, *NeST*, was found to facilitate histone methylation at the interferon gamma locus in CD8 + and Th1 cells by interacting with WDR5, a core subunit of MLL H3K4 methyltransferase (Gomez et al. 2013). All these data suggest that lncRNAs can regulate immune cell functions shedding the light on the need of studies investigating the functions of lncRNA in immune cells that directly target and kill cancer cells such as NK cells in the immune system of cancer patients. In the current study, we utilized a commercially available QPCR array of 84 lncRNAs to investigate their expression levels in circulating NK cells isolated from peripheral blood of patients with invasive breast cancer compared to healthy donors.

## Results

### Twenty six lncRNAs are differentially expressed in NK cells from breast cancer patients

**Fig. 1:**
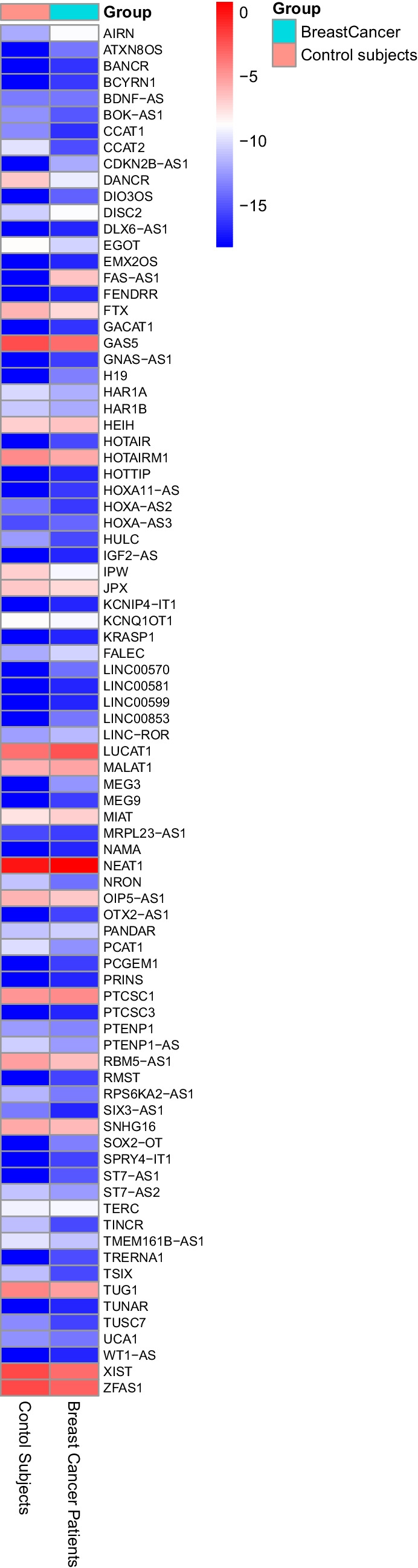
Heatmap representing the relative quantification of lncRNAs in circulating NK cells isolated from peripheral blood of breast cancer patients and control subjects. The columns represent control subjects and breast cancer patients and the rows represent the different lncRNAs. The color gradient (blue–white–red) reflects the actual average –ΔCt values, where blue indicates lower expression and red indicates higher expression. The heatmap was generated using the pheatmap package in R without clustering and reflects true differences in lncRNA expression without row-wise scaling artifacts

**Fig. 2:**
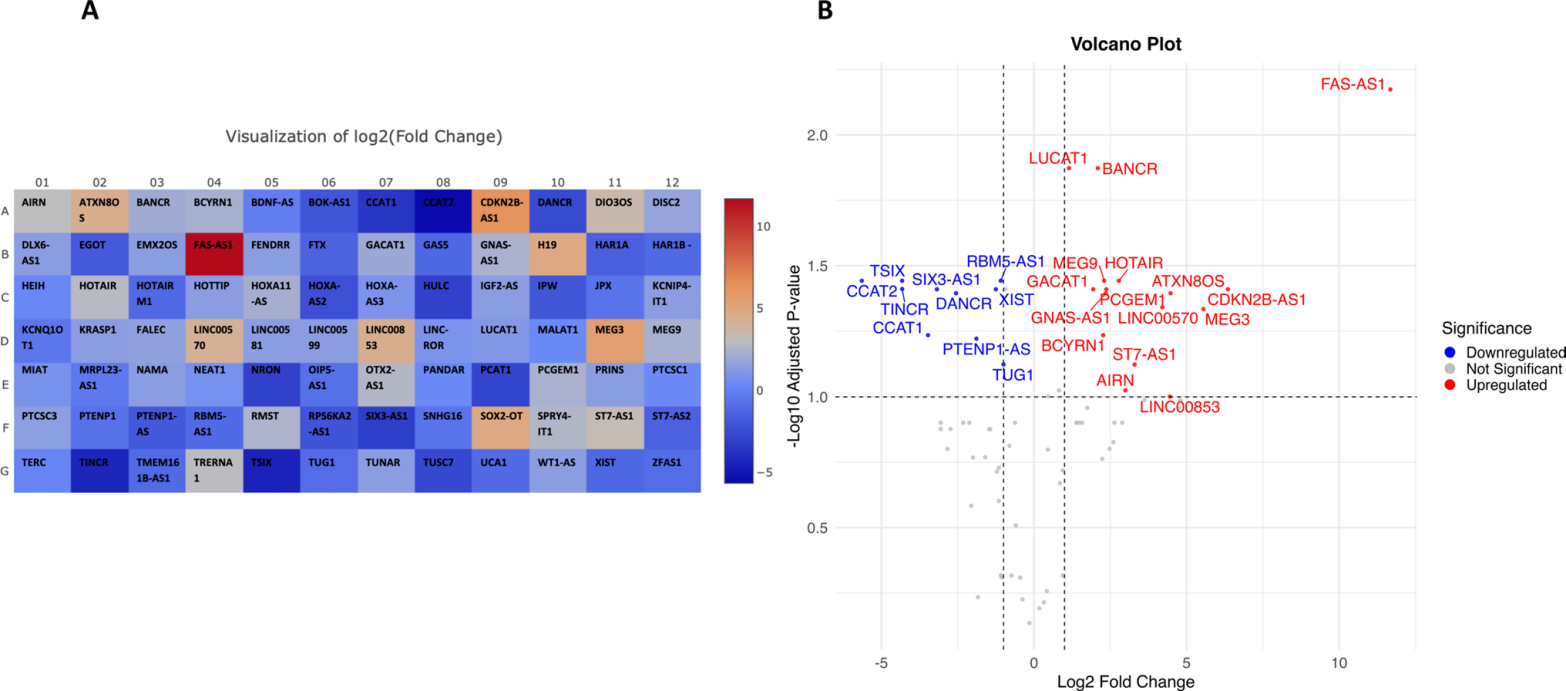
**A** A heatmap showing red and blue blocks representing increased or decreased individual lncRNAs in circulating NK cells isolated from peripheral blood of breast cancer patients against the control group (healthy donors), respectively. Each block corresponds to individual lncRNAs as per its location on the 96-well RT2 QPCR array plate, and the color intensity represents the log_2_ fold change values (2^−ΔΔCt^). **B** Volcano plot illustrating differential expression of lncRNAs in circulating NK cells isolated from peripheral blood of breast cancer patients versus healthy controls. The *x*-axis displays the log_2_ fold change (2^−ΔΔCt^), and the *y*-axis represents the –log_10_ of the adjusted *P*-values. Red and blue dots indicate significantly upregulated and downregulated lncRNAs, respectively, based on thresholds of |log_2_ fold change|≥ 1 and FDR-adjusted *P*-value < 0.1. Grey dots represent non-significant changes. Labeled genes denote those with statistical significance. The adjusted *P*-value threshold was selected after applying the two-stage step-up method of Benjamini, Krieger, and Yekutieli (BKY) for the calculation of discovery rate (FDR)-adjusted *q*-values with a significance threshold set at 10%. The heatmap in **A** was generated using the GeneGlobe Data Analysis Center (https://geneglobe.qiagen.com/eg/analyze), and the volcano plot in **B** was created in R using ggplot2 and ggrepel

**Fig. 3:**
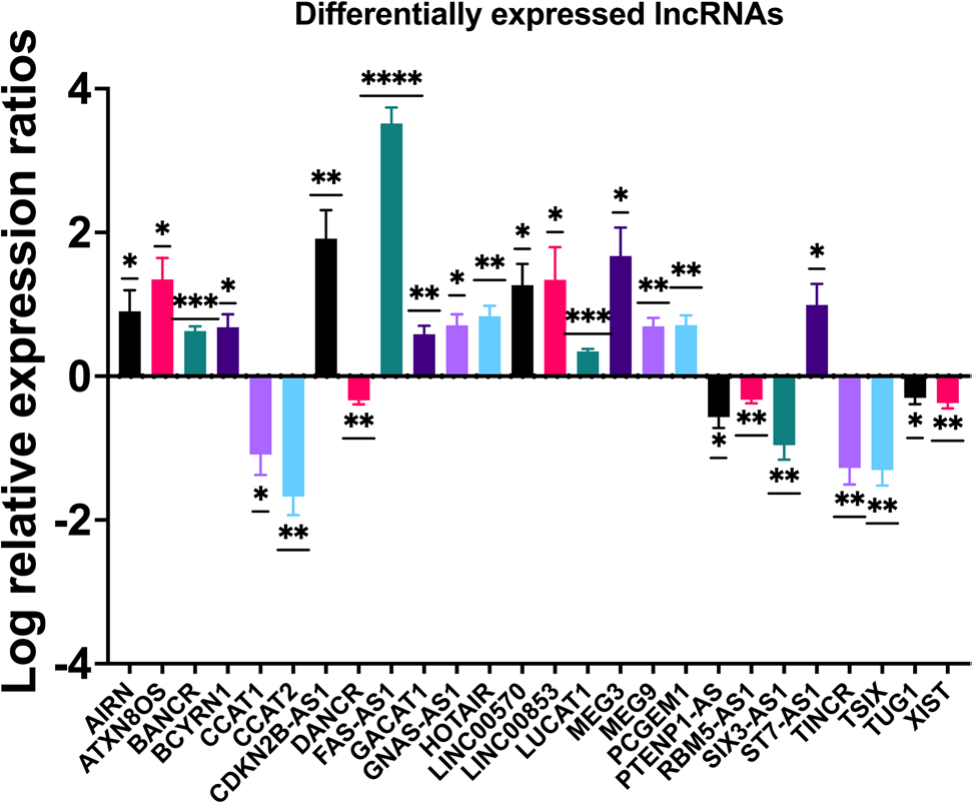
Bar chart depicting the differential expression of statistically significant DEGs in circulating NK cells isolated from peripheral blood of breast cancer patients measured as log-transformed relative expression ratios (log fold change) compared to control samples. The log transformation allows visualization of upregulation (positive values) and downregulation (negative values) of gene expression on a symmetrical scale. Each bar represents the mean ± standard error of the mean (SEM) of the log relative expression for each gene across samples. Statistical significance of expression changes was evaluated using a one-sample *t*-test against a theoretical mean of zero (indicating no change from control expression). Significance levels are indicated by asterisks: *P* < 0.05 (*), *P* < 0.01 (**), *P* < 0.001 (***), and *P* < 0.0001 (****). These annotations highlight genes whose expression significantly differs from baseline control levels

Total RNA was extracted from circulating NK cells isolated from peripheral blood of patients with invasive breast cancer and reverse transcribed into cDNA. The expression of 84 lncRNAs was done using RT^2^ QPCR array. The RT^2^ QPCR array analysis revealed a total of 26 lncRNAs differentially expressed in all breast cancer samples analyzed. Table 1 shows a list of all lncRNAs up and downregulated with fold regulation >|2| and statistically significantly dysregulated after applying the two-stage step-up method of Benjamini, Krieger, and Yekutieli (BKY) for calculation of discovery rate (FDR)-adjusted *q-*values with a significance threshold set at 10%. This is because FDR helps control for false positive genes that appear differentially expressed by chance alone. This method offers a balance between stringency and power to balance the risk of false positives with the need to detect true differences. Figure 1 presents the relative quantification of lncRNAs in circulating NK cells isolated from peripheral blood of breast cancer patients compared to normal subjects. Columns represent the two groups (normal subjects and breast cancer patients), and rows represent the differentially expressed lncRNAs. The heatmap illustrates the expression levels of different lncRNAs, with a color gradient (blue–white–red) reflecting the actual average –ΔCt values, where blue indicates lower expression and red indicates higher expression. The heatmap was generated using the pheatmap package in R without clustering and reflects true differences in lncRNA expression without row-wise scaling artifacts. Moreover, Fig. 2A shows a heatmap illustrating the differential expression of lncRNAs in circulating NK cells isolated from peripheral blood of breast cancer patients compared to healthy donors. Red and blue blocks represent increased and decreased gene expression, respectively. Figure 2B shows a volcano plot comparing the average fold change of 84 lncRNAs between the two groups, with red dots indicating upregulated genes and blue dots indicating downregulated genes. To further highlight the differentially expressed lncRNAs between breast cancer patients and control subjects, a bar chart summarizing the expression profiles of statistically significant transcripts is shown in Fig. 3. This visualization presents log-transformed relative expression values (mean ± SEM) for each gene and includes significance annotations based on one-sample *t*-tests. The chart complements the volcano plot by providing a clear view of the direction and magnitude of change in each significant lncRNA.

**Table 1:** Differentially expressed lncRNAs in circulating NK cells isolated from peripheral blood of breast cancer patients compared to healthy donors

**Unigene**	**Refseq**	**Symbol**	**Description**	**Fold change (geometric mean)**	***P-value***	q value*	**Discovery?**
Hs.742166	ENST00000601203	AIRN	Antisense of IGF2R non-protein coding RNA	7.97	0.0379	0.0944	Yes
Hs.676453	ENST00000414504	ATXN8OS	ATXN8 opposite strand (non-protein coding)	22.22	0.0108	0.0403	Yes
Hs.49768	NR_047671	BANCR	BRAF-activated non-protein coding RNA	4.26	0.0006	0.0134	Yes
N/A	ENST00000418539	BCYRN1	Brain cytoplasmic RNA 1	4.81	0.0189	0.0583	Yes
Hs.343864	ENST00000500112	CCAT1	Colon cancer associated transcript 1 (non-protein coding)	0.09	0.0191	0.0583	Yes
Hs.745578	NR_109834	CCAT2	Colon cancer associated transcript 2 (non-protein coding)	0.02	0.0028	0.0361	Yes
Hs.512599	ENST00000421632	CDKN2B-AS1	CDKN2B antisense RNA 1	81.80	0.0087	0.0389	Yes
Hs.744077	NR_024031	DANCR	KIAA0114	0.17	0.0098	0.0403	Yes
Hs.244139	NR_028371	FAS-AS1	FAS antisense RNA 1	3276.64	0.0001	0.0067	Yes
N/A	ENST00000419650	GACAT1	Gastric cancer associated transcript 1 (non-protein coding) [Source:HGNC Symbol;Acc:48336]	3.84	0.0076	0.0389	Yes
Hs.122718	NR_002785	GNAS-AS1	GNAS antisense RNA 1	5.10	0.0104	0.0403	Yes
Hs.197076	NR_003716	HOTAIR	Hox transcript antisense RNA (non-protein coding)	6.86	0.0042	0.0361	Yes
Hs.102428	ENST00000417473	LINC00570	Long intergenic non-protein coding RNA 570	18.43	0.0129	0.0456	Yes
Hs.435758	ENST00000429328	LINC00853	Long intergenic non-protein coding RNA 853	21.92	0.0419	0.0997	Yes
Hs.745403	ENST00000511918	LUCAT1	Lung cancer associated transcript 1 (non-protein coding)	2.21	0.0006	0.0134	Yes
Hs.654863	NR_002766	MEG3	Maternally expressed 3 (non-protein coding)	46.85	0.0138	0.0463	Yes
Hs.301755	ENST00000429368	MEG9	Maternally expressed 9 (non-protein coding)	4.92	0.0043	0.0361	Yes
Hs.546994	NR_002769	PCGEM1	Prostate-specific transcript 1 (non-protein coding)	5.16	0.0061	0.0389	Yes
Hs.598470	NR_103745	PTENP1-AS	PTENP1 antisense RNA	0.27	0.0206	0.0601	Yes
Hs.439480	NR_045388	RBM5-AS1	RBM5 antisense RNA 1	0.47	0.0025	0.0361	Yes
Hs.503113	ENST00000419364	SIX3-AS1	SIX3 antisense RNA 1	0.11	0.0086	0.0389	Yes
Hs.597516	ENST00000456775	ST7-AS1	ST7 antisense RNA 1	9.81	0.028	0.0752	Yes
Hs.655392	NR_027064	TINCR	Placenta-specific 2 (non-protein coding)	0.05	0.0052	0.0388	Yes
Hs.529901	NR_003255	TSIX	TSIX transcript, XIST antisense RNA (non-protein coding)	0.05	0.0037	0.0361	Yes
Hs.554829	NR_002323	TUG1	Taurine upregulated 1 (non-protein coding)	0.50	0.0276	0.0752	Yes
Hs.529901	NR_001564	XIST	X (inactive)-specific transcript (non-protein coding)	0.42	0.007	0.0389	Yes

*FDR-adjusted *q*-values with a significance threshold set at 10%

### GO and functional enrichment analysis reveal four downregulated lncRNAs: PTENP1-AS, *TSIX*, *XIST*, and CCAT1 with deposited GO terms

**Fig. 4:**
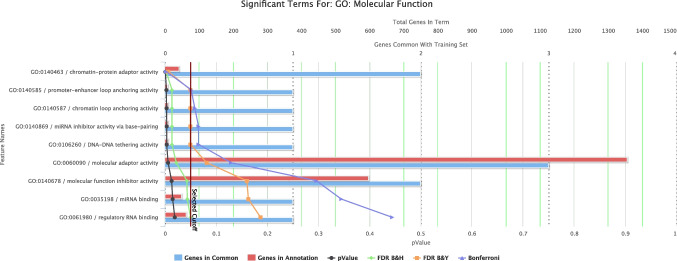
GO Molecular Function enrichment analysis of downregulated DEGs. The figure highlights the significant enrichment of the chromatin-protein adaptor activity term, identified using ToppGene analysis. Statistical significance was determined using Bonferroni correction (adjusted *P*-value < 0.05). The input genes contributing to this enrichment include *PTENP1-AS* and *XIST*. The number of genes mapped to each GO term and the corresponding Bonferroni-adjusted *P*-values are shown. Generated from https://toppgene.cchmc.org/enrichment.jsp

**Fig. 5:**
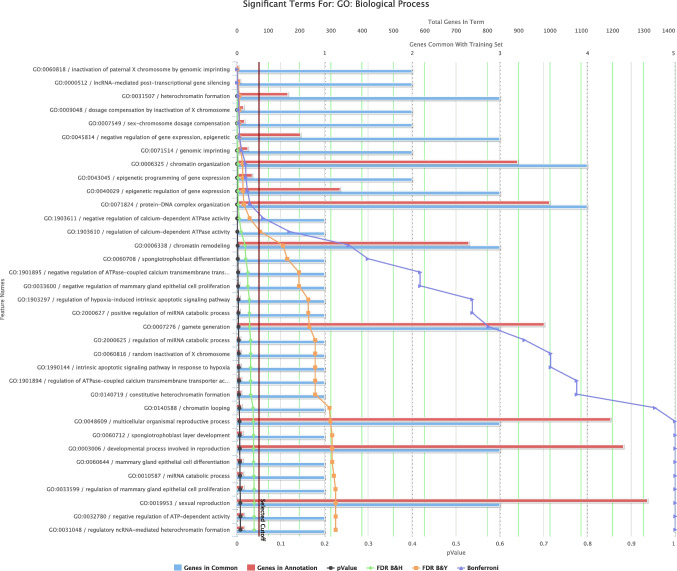
GO Biological Process enrichment analysis of downregulated DEGs. The figure presents the significantly enriched biological processes associated with downregulated DEGs, as identified by ToppGene. Prominent enriched terms include chromatin organization (*PTENP1-AS*, *XIST*, *TSIX*, and *CCAT1*) epigenetic regulation of gene expression (*PTENP1-AS*, *XIST*, and *TSIX*), lncRNA-mediated post-transcriptional gene silencing (*PTENP1-AS* and *XIST*), and dosage compensation via X chromosome inactivation (*TSIX* and *XIST*). Enrichment significance was assessed using Bonferroni correction for multiple testing, and terms with an adjusted *P*-value < 0.05 were considered significant. The number of genes mapped to each GO term and the corresponding Bonferroni-adjusted *P*-values are shown. Generated from https://toppgene.cchmc.org/enrichment.jsp

To better understand the functional relevance of these dysregulated lncRNAs, we next performed GO and functional enrichment analyses for biological process and molecular function. GO and functional enrichment analysis was done on lncRNAs downregulated with fold change < 2 showing statistical significance according to FDR-adjusted *P*-values. Using GO and functional enrichment with ToppGFun and FDR Bonferroni correction at *P*-value < 0.05 using Toppgene (Chen et al. 2009), four downregulated lncRNAs have deposited GO terms, which are *PTENP1-AS*, *FTX*, *TSIX*, *XIST*, and *CCAT1*. Figure 4 and Table 2 show the molecular functions of downregulated lncRNAs. Figure 5 and Table 3 show the biological processes of downregulated lncRNAs. The input genes contributing to chromatin-protein adaptor activity include *PTENP1-AS* and *XIST*. Prominent enriched terms under biological process include chromatin organization (*PTENP1-AS*, *XIST*, *TSIX*, and *CCAT1*), epigenetic regulation of gene expression (*PTENP1-AS*, *XIST*, and *TSIX*), lncRNA-mediated post-transcriptional gene silencing (*PTENP1-AS* and *XIST*), and dosage compensation via X chromosome inactivation (*TSIX* and *XIST*).

**Table 2:** Significantly enriched GO Molecular Function term associated with downregulated DEGs identified by ToppGene. The table lists the enriched GO Molecular Function term based on Bonferroni correction (adjusted *P*-value < 0.05), along with the corresponding *P*-value, FDR values (Benjamini–Hochberg and Benjamini-Yekutieli), Bonferroni-adjusted *P*-value, and the input genes contributing to the enrichment. Generated from https://toppgene.cchmc.org/enrichment.jsp

**ID**	**Name**	***P-value***	**FDR B&H**	**FDR B&Y**	**Bonferroni**	**Genes from input**
GO:0140463	Chromatin-protein adaptor activity	6.14E − 05	1.47E − 03	5.56E − 03	1.47E − 03	PTENP1-AS, XIST

**Table 3:** Significantly enriched GO Biological Process terms among downregulated DEGs identified by ToppGene. The table shows selected GO terms based on Bonferroni correction (adjusted *P*-value < 0.05), along with the corresponding *P*-values, FDR values (B&H and B&Y), and the input genes associated with each term. Generated from https://toppgene.cchmc.org/enrichment.jsp

**ID**	**Name**	***P-value***	**FDR B&H**	**FDR B&Y**	**Bonferroni**	**Genes from input**
GO:0060818	Inactivation of paternal X chromosome by genomic imprinting	5.92E − 07	1.04E − 04	5.99E − 04	1.04E − 04	TSIX, XIST
GO:0000512	lncRNA-mediated post-transcriptional gene silencing	4.44E − 06	3.91E − 04	2.25E − 03	7.81E − 04	PTENP1-AS, XIST
GO:0031507	Heterochromatin formation	1.66E − 05	9.37E − 04	5.39E − 03	2.92E − 03	PTENP1-AS, XIST, TSIX
GO:0009048	Dosage compensation by inactivation of X chromosome	2.27E − 05	9.37E − 04	5.39E − 03	4.00E − 03	TSIX, XIST
GO:0007549	Sex-chromosome dosage compensation	2.72E − 05	9.37E − 04	5.39E − 03	4.78E − 03	TSIX, XIST
GO:0045814	Negative regulation of gene expression, epigenetic	3.19E − 05	9.37E − 04	5.39E − 03	5.62E − 03	PTENP1-AS, XIST, TSIX
GO:0071514	Genomic imprinting	5.84E − 05	1.47E − 03	8.45E − 03	1.03E − 02	TSIX, XIST
GO:0006325	Chromatin organization	1.11E − 04	2.25E − 03	1.30E − 02	1.96E − 02	PTENP1-AS, XIST, TSIX, CCAT1
GO:0043045	Epigenetic programming of gene expression	1.15E − 04	2.25E − 03	1.30E − 02	2.03E − 02	TSIX, XIST
GO:0040029	Epigenetic regulation of gene expression	1.35E − 04	2.38E − 03	1.37E − 02	2.38E − 02	PTENP1-AS, XIST, TSIX
GO:0071824	Protein-DNA complex organization	1.70E − 04	2.72E − 03	1.57E − 02	3.00E − 02	PTENP1-AS, XIST, TSIX, CCAT1

### GO and functional enrichment analysis reveals 5 upregulated lncRNAs*: GNAS-AS1*, *MEG3*, *CDKN2B-AS1*, *HOTAIR*, and *AIRN*, with deposited GO terms

**Fig. 6:**
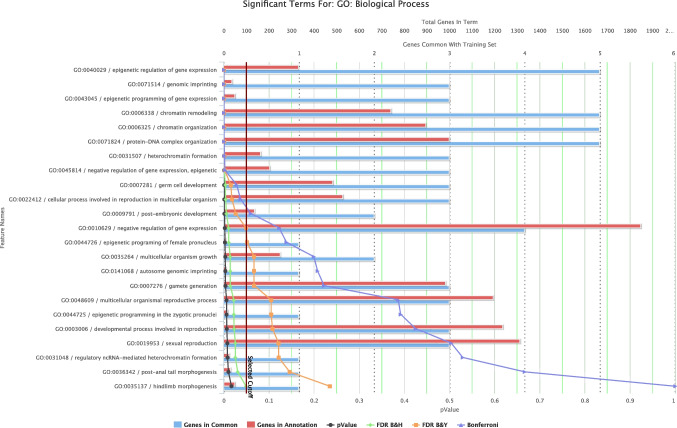
GO Biological Process enrichment analysis of upregulated DEGs. The figure displays the significantly enriched biological processes identified by ToppGene among upregulated DEGs. Prominent enriched terms include epigenetic regulation of gene expression (*CDKN2B-AS1*, *HOTAIR*, *GNAS-AS1*, *MEG3*, and *AIRN*), genomic imprinting (*GNAS-AS1*, *MEG3*, and *AIRN*), chromatin remodeling *CDKN2B-AS1*, *HOTAIR*, *GNAS-AS1*, *MEG3*, and *AIRN*, and protein-DNA complex organization *CDKN2B-AS1*, *HOTAIR*, *GNAS-AS1*, *MEG3*, and *AIRN*. Significance was assessed using Bonferroni correction (adjusted *P*-value < 0.05), and the number of genes associated with each biological process is indicated. Generated from https://toppgene.cchmc.org/enrichment.jsp

Similarly, we performed GO and functional enrichment analyses for biological processes and molecular functions using the ToppGene tool using ToppGFun (Chen et al. 2009). GO and functional enrichment analysis were done on lncRNAs upregulated with fold change > 2 and showing statistical significance according to FDR-adjusted *P*-values. The analysis revealed no matching hits for molecular functions. For biological processes, however, the analysis revealed five upregulated lncRNAs with deposited significantly enriched GO terms (Bonferroni FDR correction at *P-*value < 0.05). These lncRNAs are *GNAS-AS1*, *MEG3*, *CDKN2B-AS1*, *HOTAIR*, and *AIRN*. Prominent enriched terms include epigenetic regulation of gene expression (*CDKN2B-AS1*, *HOTAIR*, *GNAS-AS1*, *MEG3*, and *AIRN*), genomic imprinting (*GNAS-AS1*, *MEG3*, and *AIRN*), chromatin remodeling (*CDKN2B-AS1*, *HOTAIR*, *GNAS-AS1*, *MEG3*, and *AIRN*), and protein-DNA complex organization (*CDKN2B-AS1*, *HOTAIR*, *GNAS-AS1*, *MEG3*, and *AIRN*). Table 4 and Fig. 6 show the biological processes of upregulated lncRNAs.

**Table 4:** Significantly enriched GO Biological Process terms associated with upregulated DEGs. The table lists the top GO Biological Process terms identified through ToppGene analysis among upregulated DEGs. Enrichment significance was determined using Bonferroni correction (adjusted *P*-value < 0.05). Terms related to epigenetic regulation, chromatin organization, and genomic imprinting were among the most significantly enriched. For each term, the *P*-value, FDR values (Benjamini–Hochberg and Benjamini-Yekutieli), Bonferroni-adjusted *P*-value, and the corresponding input genes are indicated. Generated from https://toppgene.cchmc.org/enrichment.jsp

**ID**	**Name**	*P-*value	FDR B&H	FDR B&Y	Bonferroni	Genes from input
GO:0040029	Epigenetic regulation of gene expression	2.08E − 08	1.42E − 06	6.80E − 06	1.42E − 06	CDKN2B-AS1, HOTAIR, GNAS-AS1, MEG3, AIRN
GO:0071514	Genomic imprinting	1.56E − 07	5.30E − 06	2.55E − 05	1.06E − 05	GNAS-AS1, MEG3, AIRN
GO:0043045	Epigenetic programming of gene expression	4.38E − 07	9.93E − 06	4.77E − 05	2.98E − 05	GNAS-AS1, MEG3, AIRN
GO:0006338	Chromatin remodeling	1.17E − 06	1.99E − 05	9.54E − 05	7.94E − 05	CDKN2B-AS1, HOTAIR, GNAS-AS1, MEG3, AIRN
GO:0006325	Chromatin organization	2.99E − 06	4.06E − 05	1.95E − 04	2.03E − 04	CDKN2B-AS1, HOTAIR, GNAS-AS1, MEG3, AIRN
GO:0071824	Protein-DNA complex organization	5.11E − 06	5.79E − 05	2.78E − 04	3.47E − 04	CDKN2B-AS1, HOTAIR, GNAS-AS1, MEG3, AIRN
GO:0031507	Heterochromatin formation	1.66E − 05	1.61E − 04	7.73E − 04	1.13E − 03	CDKN2B-AS1, HOTAIR, AIRN
GO:0045814	Negative regulation of gene expression, epigenetic	3.19E − 05	2.71E − 04	1.30E − 03	2.17E − 03	CDKN2B-AS1, HOTAIR, AIRN
GO:0007281	Germ cell development	4.14E − 04	3.13E − 03	1.50E − 02	2.81E − 02	GNAS-AS1, MEG3, AIRN
GO:0022412	Cellular process involved in reproduction in multicellular organism	5.38E − 04	3.66E − 03	1.76E − 02	3.66E − 02	GNAS-AS1, MEG3, AIRN

## Discussion

Given the antitumor immune responses that NK cells have, it is pivotal to study their regulation in the immune system of cancer patients. lncRNAs have now proven roles in regulating gene expression at the transcriptional as well as post-transcriptional levels. The current study aims at simultaneously analyzing the expression of 84 lncRNAs using RT^2^ PCR arrays in circulating NK cells isolated from peripheral blood of patients with breast cancer. Gene expression analysis revealed differential expression of 26 lncRNAs in circulating NK cells isolated from peripheral blood of breast cancer patients compared to healthy controls. Of these 26 differentially expressed lncRNAs, gene ontology and functional enrichment analysis using Toppgene (Chen et al. 2009), four downregulated lncRNAs have deposited GO terms which are *TSIX*, *XIST*, *PTENP1-AS*, and *CCAT1.* On the other hand, five upregulated lncRNAs that have deposited GO terms which are *GNAS-AS1*, *MEG3*, *CDKN2B-AS1*, *HOTAIR*, and *AIRN*.

Here, we show that *XIST* is among the downregulated lncRNAs in circulating NK cells isolated from peripheral blood of breast cancer patients with fold change 0.42, *P* = 0.007. *XIST* is functionally annotated to gene ontology terms of biological process: “epigenetic regulation of gene expression” as well as “dosage compensation by inactivation of X chromosome.” *XIST*was also functionally annotated to the GO term of molecular function “chromatin protein adaptor activity,” “regulatory RNA binding,” as well as “molecular adaptor activity.” *XIST* is the first long non-coding gene identified within the X inactivation center (XIC). Contrary to breast cancer, She et al. (2022) reported increased expression of *XIST* in NK cells in the autoimmune disease primary biliary cholangitis (She et al. 2022) promoting NK cell proliferation. Here, we suggest that decreased expression of *XIST* in circulating NK cells isolated from peripheral blood of breast cancer patients can decrease NK cell proliferation, reducing NK cell counts in breast cancer patients. Zhao et. al (2021) reported the role of *XIST* in regulating immune cells, including macrophages, in the tumor microenvironment by the *Xist*/miR-101-3p/KLF6/C/EBPα, which might extend to NK cells through similar epigenetic and gene regulatory mechanisms (Zhao et al. 2021).

*TSIX* was also among the downregulated lncRNAs in circulating NK cells isolated from peripheral blood of breast cancer patients with fold change 0.05, *P* = 0.0037. *TSIX* gene is an antisense gene to *XIST* at the XIC and also plays a role in X chromosome inactivation (Lee et al. 1999). Downregulation of *TSIX* suggests that circulating NK cells in breast cancer patients have dysregulated XIC compared to healthy controls. Given the epigenetic mechanisms of gene regulation that *TSIX* and *XIST* play in immune cells, their dysregulation can affect NK cell activities in cancer patients, such as NK cell cytotoxicity and cytokine release.

Another lncRNA that was found to be downregulated in circulating NK cells isolated from peripheral blood of breast cancer patients is *CCAT1* with fold change 0.09, *P* = 0.0191. *CCAT1* was functionally annotated to the GO terms “chromatin organization” and “protein-DNA complex organization.” *CCAT1* was originally discovered to be overexpressed in colorectal carcinoma (Nissan et al. 2012) as well as a number of other types of cancers (Liau et al. 2023). *CCAT1* gene is located on human chromosome 8q24 (chr.8q24) region, specifically 8q24.21 nearby the *c-MYC* oncogene (Liu et al. 2019a). A significant body of evidence showed that *CCAT1* can regulate miRNA functions by the binding of miRNA response element (MRE) located at the 3’ region of *CCAT1* to the seed region of miRNAs (Chen et al. 2016; Liu et al. 2019b). Previously, it was shown in colon cancer cells, downregulation of *CCAT1* upregulated the expression of cyclin-dependent kinase inhibitor 1 A (CDKN1A) mRNA. CDKN1A controls G1 cell cycle arrest, resulting in decreased cellular proliferation (Kim et al. 2014; Luo et al. 2014). Similarly, downregulation of *CCAT1* in NK cells can result in decreased NK cell proliferation, further explaining NK cell functional deficiency in breast cancer patients. Moreover, it was shown that downregulation of *CCAT1* resulted in increased expression of proapoptotic protein Bcl-2-associated X protein (BAX) by p53 signaling cascade, resulting in induction of apoptosis of colorectal carcinoma cells. Likewise, in NK cells, downregulation of *CCAT1* in NK cells of breast cancer patients has the potential to induce apoptosis in NK cells, further explaining the functional deficiency of NK cells in breast cancer patients. Xiang et al. (2014) showed that *CCAT1* regulates MYC transcription by promoting long-range chromatin looping. Their study showed that knockdown of *CCAT1* reduced the interaction between the MYC promoter and its enhancers, highlighting its role in MYC transcriptional regulation (Xiang et al. 2014).

Khameneh et al. (2023) demonstrated that knock-out of *Myc* in mice presented a significant reduction in NK cell numbers. Khameneh et al. (2023) also showed that Myc protein is essential for NK cell development and proliferation in response to IL-15. The same group also obtained similar results in human primary NK cells. Khameneh et al. (2023) also demonstrated that in *Myc* knock-out mice, NK cells showed defective antitumor immunity, with reduced numbers of mature NK cells and impaired cytotoxic function (Khameneh et al. 2023). In line with these findings, Tang et al. (2017) employed gain-of-function and loss-of-function experiments on mice and demonstrated that Myc is essential for NK cell development, proliferation, and tumor surveillance (Tang et al. 2017). Taken together, reduced *CCAT1* expression has the potential to reduce the transcription of *MYC* gene, thereby having the potential to impair NK cell functions in cancer patients.

*PTENP1-AS* was significantly downregulated in circulating NK cells isolated from peripheral blood of breast cancer patients (fold change 0.27, *P* = 0.0206). As a transcriptional suppressor, *PTENP1-AS* promotes epigenetic silencing of *PTEN* by recruiting EZH2 and DNMT3A to the *PTEN* promoter (Johnsson et al. 2013). *PTENP1-AS* downregulation is expected to increase *PTEN* expression and attenuate PI3K/AKT/mTOR signaling (Glaviano et al. 2023). Dysregulation of PI3K/AKT/mTOR has the potential to affect NK cell granule polarization, Ca^++^ influx, cytokine production, and synapse formation (Chen et al. 2024). This aligns with the fact that elevated *PTEN *expression has been reported to impair NK cell cytotoxic function by disrupting the formation and organization of the NK cell immune synapse (Briercheck et al. 2015).

Among the upregulated lncRNAs in circulating NK cells isolated from peripheral blood of breast cancer patients is *HOTAIR* with fold change 6.86, *P* = 0.0042. In fact, *HOTAIR* is the first characterized lncRNA and was shown to interact with the polycomb repressive complex 2 (PRC2) which plays an important role in chromatin remodeling, thereby regulating gene expression (Rinn et al. 2007). Li et. al (2017) have demonstrated that chromatin state dynamics have a significant effect on NK cell activation and provided evidence that the regulation of NK cellular cytotoxic potential and immunoregulatory functions is dependent on the status of histone modification (Li et al. 2017). PRC2 represses gene transcription by introducing a trimethyl group at lysine 27 on histone 3 (H3K27me3) (Vijayanathan et al. 2022). A core methyltransferase component of the PRC2 is Enhancer of Zeste Homolog 2 (EZH2) (Sun et al. 2022). Several studies reported that EZH2 inhibition or deletion enhances the antitumor activity of NK cells by enhancing NK cell cytotoxicity and cytokine production of IFN-γ (Yin et al. 2015; Yu et al. 2021). Taken together, by acting as a scaffold and interacting with the PRC2 complex, the upregulation of *HOTAIR* in NK cells from breast cancer patients can negatively affect the antitumor immunity of NK cells in breast cancer patients.

*AIRN*, a genomically imprinted lncRNA, is another lncRNA that is overexpressed in circulating NK cells isolated from peripheral blood of breast cancer patients with fold change of 7.97, *P* = 0.0379. Similar to *HOTAIR*, *AIRN* recruits the histone modifying complex PRC2 (Andergassen et al. 2019) potentially mediating gene silencing in NK cells and affecting its antitumor activity in breast cancer patients.

*CDKN2B-AS1* was found to be upregulated in circulating NK cells isolated from peripheral blood of breast cancer patients with fold change 81.80, *P* = 0.0087. CDKN2B-AS1 gene is located within the *CDKN2B-CDKN2A* gene cluster at chromosome 9p21 (Ozuynuk-Ertugrul et al. 2024). Similarly, *CDKN2B-AS1* was shown to interact with PRC1 and PRC2, leading to epigenetic silencing of other genes in this cluster (Sanchez et al. 2023). Huang et al. 2023) have shown that *CDKN2B-AS1* RNA sponges miR-181a in granulosa cells (Huang et al. 2023). Previously, our lab demonstrated that circulating NK cells from breast cancer patients exhibited downregulation of miR-181a (Rady et al. 2017). Therefore, increased expression of *CDKN2B-AS1* can provide an explanation of the reduced expression of its miRNA target, miR-181a, in circulating NK cells from breast cancer patients.

*MEG3*, a genomically imprinted gene that was found to be significantly upregulated in circulating NK cells isolated from peripheral blood of breast cancer patients with fold change 46.85, *P* = 0.0138. Previously, it was shown that immune cell markers in tumor infiltrating immune cells including NK cells negatively correlated with the level expression of *MEG3* in glioma microenvironment suggesting that *MEG3* plays an important role in immune escape in the glioma microenvironment (Xu et al. 2021). *MEG3* is also a chromatin interacting lncRNA that interacts with PRC2 by formation of RNA–DNA triplex in homopolypurine GA-rich sequences, therefore, guiding *MEG3* RNA to its target genes (Mondal et al. 2015). *MEG3*/PRC2 interaction was shown to regulate TGF-β pathway genes (Mondal et al. 2015). Mondal et al. (2015) showed that downregulation of *MEG3* in BT-549 cells led to upregulation of *TGFB2*, *TGFBR1*, and *SMAD2* genes, known to regulate multiple cellular processes, such as cell proliferation, apoptosis, and differentiation.

**Fig. 7:**
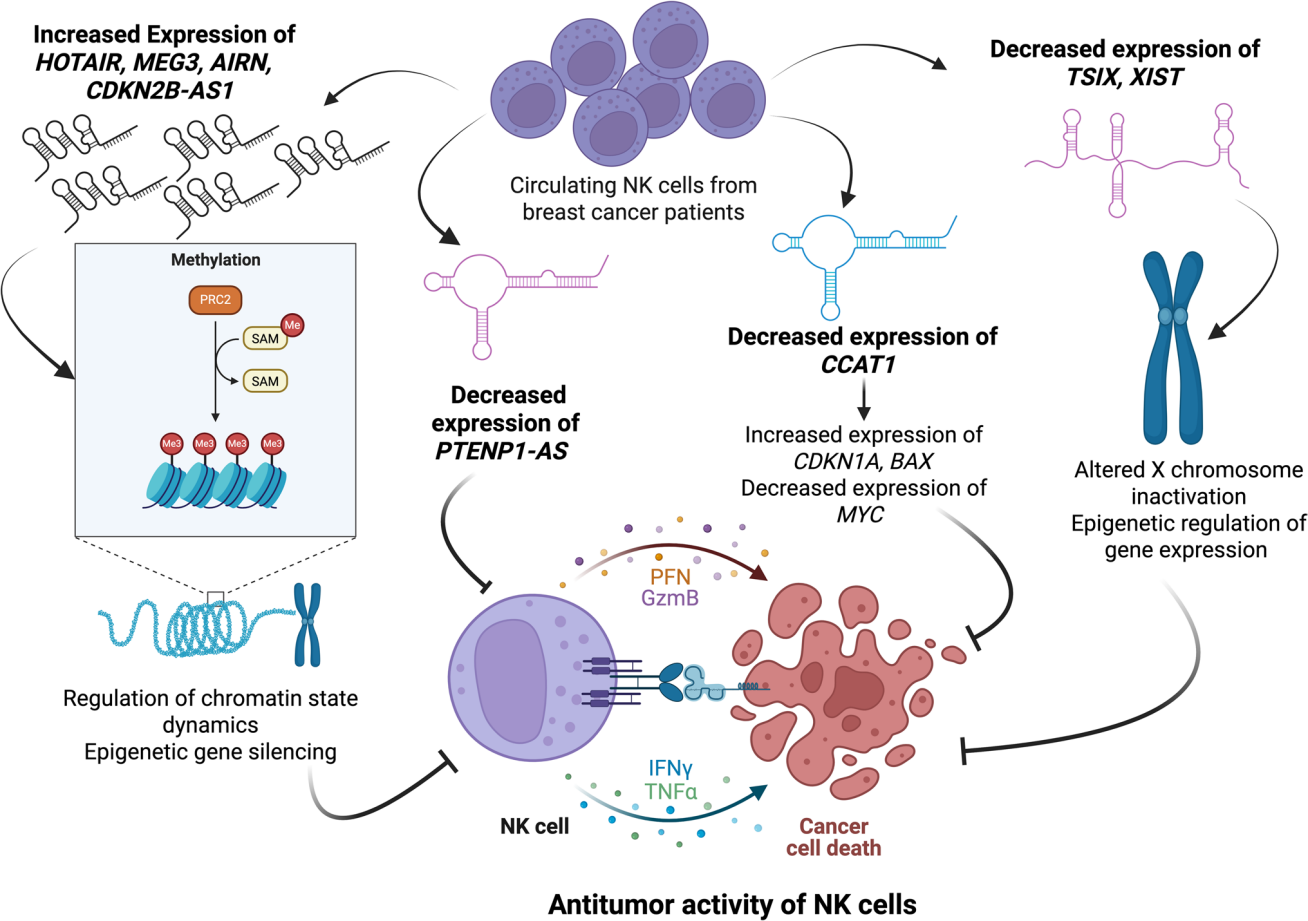
Dysregulated lncRNAs and their potential impact on NK cell function in breast cancer. This diagram summarizes altered lncRNA expression in breast cancer-associated NK cells and their hypothesized roles in modulating NK cell activity through epigenetic, transcriptional, and post-transcriptional mechanisms. T-shaped arrows (⊣) denote inhibition. Solid arrows (→) connect dysregulated lncRNAs to their direct molecular functions or their downstream impacts on NK cell activity (e.g., cytotoxicity, cytokine production). *XIST* and *TSIX* were significantly downregulated, suggesting dysregulation of X chromosome inactivation machinery, potentially affecting NK cell proliferation and survival. *CCAT1* downregulation may impair NK cell proliferation and survival via increased *CDKN1A* and *BAX* expression and reduced *MYC* transcription, crucial for NK cell development and tumor surveillance. *PTENP1-AS* downregulation could elevate *PTEN* levels, disrupting PI3K/AKT/mTOR signaling, critical for NK cell cytotoxicity and synapse formation. *HOTAIR*, *AIRN*, *CDKN2B-AS1*, and *MEG3* were upregulated and are known to recruit chromatin remodeling complexes (e.g., PRC2) potentially leading to epigenetic gene silencing, impaired NK cell activation, reduced IFN-γ production, and altered degranulation processes. This model is speculative and intended to guide future mechanistic investigations into how lncRNA dysregulation may contribute to NK cell dysfunction in cancer. Created in https://BioRender.com

*GNAS-AS1* was found to be upregulated in circulating NK cells isolated from peripheral blood of breast cancer patients with fold change 5.10, *P* = 0.0104. Liu et al. (2020) demonstrated that *GNAS-AS1* acts as a molecular sponge of miR-433-3p regulating the expression of the zinc-finger transcription factor, *GATA3* in breast cancer (Liu et al. 2020). Ali et al. (2016) have shown that GATA3 is required for IFN-γ production by NK cells (Ali et al. 2016). Also, by sponging miR-433-3p, *GNAS-AS1* regulates the expression of the GTP-binding protein, RAB3A (He et al. 2023). By serving as molecular switches, GTP-binding proteins regulate NK cell degranulation and cytotoxicity (Khurana and Leibson 2003; Maghazachi 2003; Sánchez-Ruiz et al. 2011). Figure 7 summarizes the potential impact of lncRNA dysregulation in NK cell functions in breast cancer. Taken together, the dysregulated expression of the aforementioned lncRNAs in circulating NK cells isolated from peripheral blood of breast cancer patients—and given their biological functions—can explain the NK cell immune defects in breast cancer patients (Caras et al. 2004).

Although *GAS5* or growth arrest specific 5 lncRNA was downregulated in circulating NK cells isolated from peripheral blood of breast cancer patients with fold change of 0.43, this difference was, however, statistically insignificant (*P* = 0.1984). Previously, Fang et al. (2019) studied the role of the lncRNA *GAS5* in regulating NK cell functions in liver cancer patients. Similar to our findings, expression analysis revealed that *GAS5* is downregulated in NK cells isolated from patients with hepatocellular carcinoma (Fang et al. 2019). Moreover, upon IL2 stimulation of NK-92 cells and human primary NK cells, *GAS5* expression was upregulated, suggesting a role of *GAS5* in regulating NK cell functions. Indeed, the knockdown of *GAS5* in primary human NK cells reduced NK cell cytotoxicity, degranulation as well as IFN-γ production (Fang et al. 2019). Moreover, Fang et. al (2019) showed that *GAS5* overexpression promoted the killing effect of NK cells on liver cancer through regulating miR-544/RUNX3 (Fang et al. 2019). Similarly, NK cells isolated from patients with gastric carcinoma showed downregulation of *GAS5* compared to healthy controls (Wei et al. 2020). Wei et al. (2020) also showed that *GAS5* expression was upregulated upon stimulation of either primary NK cells or the NK-92 cell line with IL2, suggesting that *GAS5* is upregulated in activated NK cells. The knockdown of *GAS5* using siRNAs in human primary NK cells and NK-92 cells resulted in reduced NK cell cytotoxicity as well as IFN-γ and TNF-α release (Wei et al. 2020) Using RNA immunoprecipitation and RNA pulldown assay, Wei et al. (2020) confirmed the interaction between miR-18a and *GAS5* in NK cells. By acting as a molecular sponge for miR-18a, *GAS5* has the potential to promote NK cell cytotoxicity.

One of the key limitations of our study is that it is restricted to the expression profiling of lncRNAs in circulating NK cells isolated from peripheral blood of breast cancer patients, without accompanying functional validation. While we identified differential expression patterns of specific lncRNAs, we did not assess the downstream effects of these lncRNAs on NK cell cytotoxicity, cytokine production, or receptor expression. As such, the direct impact of the observed lncRNA alterations on NK cell function remains speculative. Future studies incorporating functional assays and mechanistic investigations are necessary to elucidate the biological relevance of these lncRNAs in modulating NK cell activity in the context of breast cancer.

In conclusion, our findings provide compelling evidence that circulating NK cells isolated from peripheral blood of breast cancer patients exhibit dysregulated expression of a substantial number of lncRNAs. This aberrant lncRNA expression profile potentially has a significant impact on the antitumor functionality of NK cells, potentially contributing to the impaired immune surveillance and tumor control observed in breast cancer patients. The intricate interplay between these lncRNAs and NK cell functionality underscores the need for further research to elucidate the functional roles of specific lncRNAs in primary NK cells. Comprehensive functional analyses are essential to determine how these lncRNAs modulate NK cell activities and to assess their potential to modulate NK cell cytotoxicity or cytokine production in breast cancer patients. Such studies will enhance our understanding of the molecular mechanisms underpinning NK cell dysfunction in breast cancer and could identify novel immunotherapeutic strategies aimed at restoring effective antitumor immunity.

## Materials and methods

### Ethics

All experimental protocols were approved by the local ethics committees of the NCI, Cairo University and the German University in Cairo and conducted according to their guidelines and regulations and according to the Declaration of Helsinki. Informed consents were obtained from breast cancer patients and healthy donors in accordance with the Declaration of Helsinki.

### Patients and blood samples

Peripheral blood samples were procured from 19 patients admitted to the National Cancer Institute (NCI), Cairo University, with a histologically confirmed diagnosis of invasive breast cancer. All female breast cancer patient samples included in this study were collected at the time of initial diagnosis, prior to the initiation of any form of chemotherapy, radiotherapy, or immunotherapy. Patients were grouped into five groups based on the expression of estrogen receptor, progesterone receptor, and HER-2/neu into TNBC, Luminal A, Luminal B HER2 +, Luminal B HER2-, and HER2-enriched. Peripheral blood samples were also procured from five healthy female donors. Exclusion criteria for both breast cancer patients and healthy donors included any concurrent disease condition, which can cause chronic inflammation. Table 5 shows the clinical data of breast cancer patients and healthy donors.

**Table 5:** Clinical data of breast cancer patients and healthy controls included in the study

	**Number**	**Median age (years)**
**Group characteristic**		
Healthy group	5	41
Breast cancer group	19	44.5
**Breast cancer molecur subtype**		
Luminal A	7	
Luminal B HER2 +	5	
Luminal B HER2-	1	
HER2-enriched	5	
TNBC	1	

### NK cell isolation

Peripheral blood of patients with breast cancer and healthy donors was collected in heparin vacutainers, BD Biosciences, San Jose, CA, USA. The lymphocyte separation medium, Lonza Walkersville, Inc., Houston TX, USA, was used for the separation of peripheral blood mononuclear cells (PBMCs) from peripheral blood samples. The PBMCs’ cell pellets were washed twice in isolation buffer; Dulbecco’s PBS buffer without calcium or magnesium, containing 2% FCS and 2 mM EDTA. Cell counts were done using a hemocytometer and trypan blue staining. Cells were suspended at 5 × 10^7^/500 µl isolation buffer. NK cells were isolated using immunomagnetic negative selection using Dynabeads® Untouched™ Human NK Cells kit, Invitrogen Cergy-Pontoise, France, following the manufacturer’s recommendations. Flow cytometry analysis confirmed that the isolated NK cells were highly pure, consisting of over 95% CD3⁻CD56⁺ cells and less than 5% CD3⁺ contamination (Supplementary Fig. 1).

### Total RNA isolation

Following NK cell isolation, 1 ml TRIzol reagent (Invitrogen Cergy-Pontoise, France) was added to NK cells for total RNA extraction following the manufacturer’s recommendations. Spectrophotometric analysis of extracted RNA was done using a NanoDrop 2000 UV–Vis spectrophotometer (Thermo Scientific, UK) at *λ*_260_ nm and *λ*_280_ nm to assess the RNA concentration and purity. The A260/A280 ratios were between 1.7 and 2.1 for all samples. Total RNA integrity was assessed by running total RNA on 1% agarose gel electrophoresis. Intact RNA had two clearly visible 28S and 18S rRNA bands (Supplementary Fig.  2A). The integrity of total RNA was further evaluated using a denaturing 15% polyacrylamide gel, with visible bands for tRNA, 5S rRNA, and 5.8S rRNA, indicating intact RNA quality. This is relevant for lncRNAs that fall at the lower end of the size spectrum or overlap in size with small RNAs (Supplementary Fig. 2B).

### Reverse transcription and RT^2^ QPCR lncRNA PCR array

To ensure comprehensive analysis of gene expression profiles across diverse breast cancer subtypes, RNA samples from patients representing all five molecular subgroups (Luminal A, Luminal B HER2-, Luminal B HER2 +, HER2-Enriched, and Triple-Negative) were pooled for use in QPCR arrays, allowing for a robust comparison of target gene expression patterns while minimizing individual patient variability. Reverse transcription was done using 1 µg total RNA as template using the RT2 First strand kit (Qiagen, Hilden, Germany) in a final reaction volume of 20 µl and according to the manufacturer’s directions. The reverse transcription mix was completed to 111 µl. The PCR reaction mixes were prepared in RT2 PCR Array Loading Reservoir (Qiagen, Hilden, Germany). The product of cDNA synthesis reaction of a volume of 102 µl was added to 1350 µl of RT2 SYBR Green ROX qPCR Mastermix, Qiagen, Hilden Germany and completed to a final reaction volume of 2700 µl with nuclease-free water. The PCR component mix was dispensed into the RT2 IncRNA PCR Array format A (catalogue # LAHS-001ZA-6). QPCR was performed using MX3005 P™ quantitative real-time PCR system (Stratagene, La Jolla, San Diego, California, USA). The following thermal profile was used: 95 °C for 10 min, followed by 40 cycles of 95 °C for 15 s and 60 °C for 1 min. A a dissociation curve was run at the end of each PCR run using the following thermal profile: 95 °C for 1 min, 55 °C for 30 s, 95 °C for 30 s to test for the specificity of each assay.

### RT^2^ QPCR profiler data analysis

The data analysis was performed with MxPro QPCR software version 4.01 (Stratagene, La Jolla, San Diego, California, USA). The threshold was adjusted manually to be having the same value across all RT2 lncRNA array runs so that it is above the background signal but within the lower third of the linear phase of the amplification plot by using the log view of the amplification plots. The stratagene MxPro QPCR software version 4.01 allows the fluorescence baseline to be automatically adjusted using the adaptive baseline function. The cycle threshold (Ct) values were generated individually for each breast cancer patients’ group and healthy donors’ group for each target lncRNA gene. The Ct values for all wells were exported to a blank Excel spreadsheet. Data analysis was conducted using QIAGEN’S GeneGlobe Data Analysis Center available at https://geneglobe.qiagen.com/eg/analyze. The five reference genes ACTB, B2M, RPLP0, RN7SK, and SNORA73A were used to normalize relative expression ratios by calculating the 2^(−ΔΔCt)^ for each lncRNA gene in the plate (Livak and Schmittgen 2001). Genes were considered differentially expressed if they exhibited a fold change ≥ 2 or ≤ 0.5. In addition to the fold-change threshold, statistical significance was assessed using the two-stage step-up method of Benjamini, Krieger, and Yekutieli (BKY) to calculate discovery rate (FDR)-adjusted *P*-values, with a significance threshold set at 10%. The FDR method was applied to control for the potential occurrence of false positives due to multiple comparisons, offering a balance between statistical stringency and the ability to detect true differences in gene expression. Only genes meeting both the fold-change and FDR-adjusted *P*-value criteria were considered significantly differentially expressed.

### Gene ontology and functional enrichment analysis

Gene list functional enrichment was done using ToppGene Suite (Chen et al. 2009) available at: https://toppgene.cchmc.org. ToppFun detects functional enrichment of input gene list based on gene expression. Two lists of genes were analyzed: upregulated differentially expressed genes (DEGs) and downregulated DEGs. We performed the gene enrichment analysis for biological processes and molecular functions with ToppGFun and FDR correction at *P*-value < 0.05. Only statistically significant GO terms set above the cut-off and the Bonferroni-adjusted *P*-values were considered.

## Supplementary Information

Below is the link to the electronic supplementary material.

ESM 1 DOCX (467 KB)

## Data Availability

The raw QPCR data are available upon request from the corresponding author.
