# Supplementary material for: Dysregulation of lncRNAs in NK cells from breast cancer patients: implications for NK cell functions
Source: Immunogenetics. 2025 Aug 9;77(1):26. doi: 10.1007/s00251-025-01383-x (PMC12334489; doi:10.1007/s00251-025-01383-x)
Supplement: Supplementary file 1 — DOCX (467 KB) [file 251_2025_1383_MOESM1_ESM.docx]

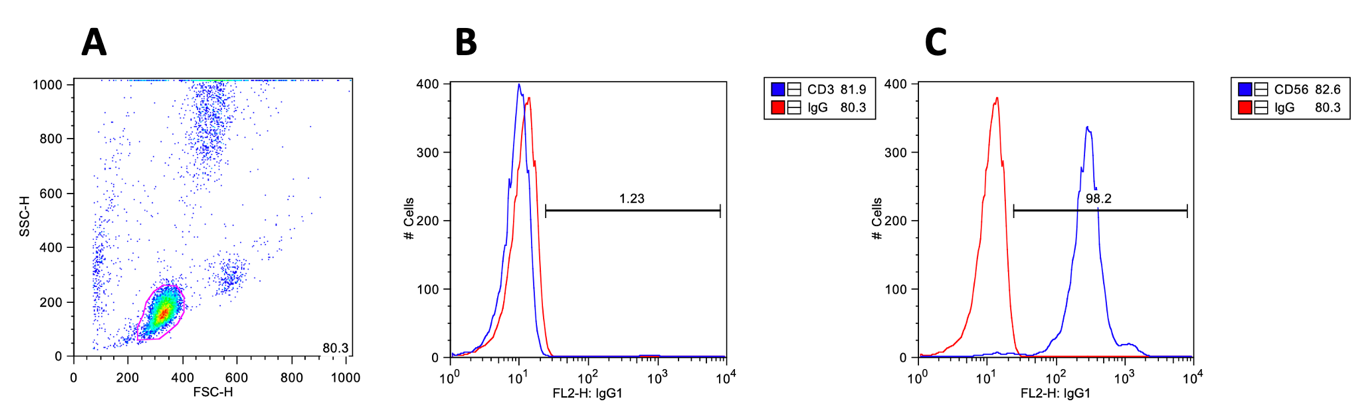


Supplementary figure 1: FACS analysis of NK cells isolated from peripheral blood samples. Within the lymphocyte gate (A), 1.23% of the cells were positive for CD3 (B) and 98.2% of the cells were positive for CD56 (C).


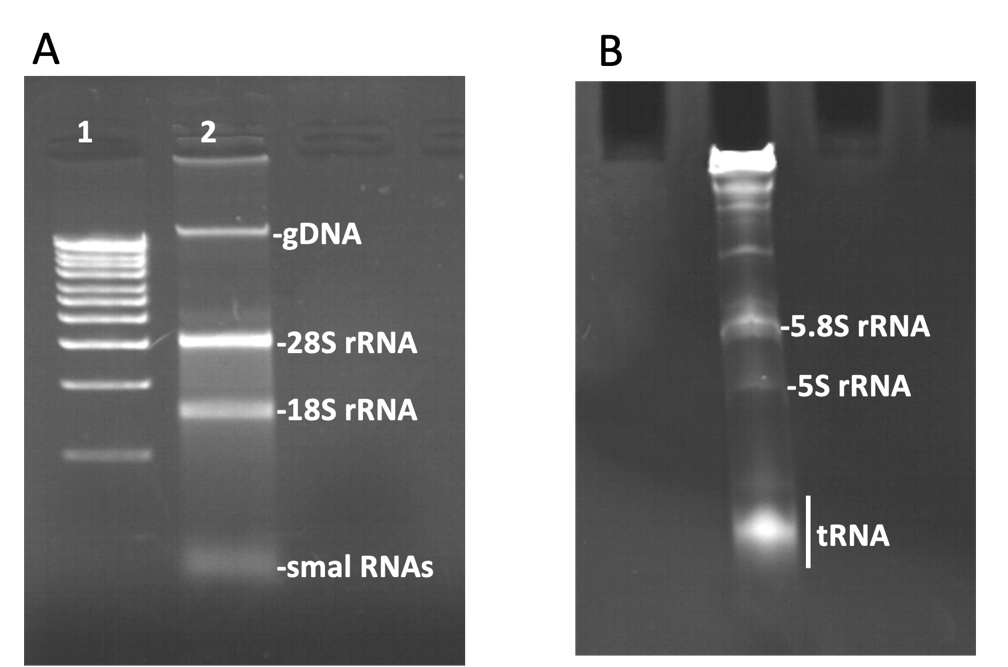


Supplementary figure 2: **A:** Agarose gel electrophoresis and ethidium bromide staining was used to check the integrity of total RNA. Total RNA run on an 1 % agarose gel shows sharp 28S and 18S rRNA bands at 4.8 kb and 1.8 kb, respectively. The 28S rRNA band is approximately twice as intense as the 18S rRNA band. This 2:1 ratio (28S:18S) is the indication that the RNA is intact. Lane 1: ladder. Lane 2: total RNA isolated from NK cells. **B:** Total RNA run on 15% denaturing polyacrylamide gel stained with ethidium bromide. Visible tRNA (~70—90 bp), 5S rRNA (~120 bp), and 5.8S rRNA (~160 bp) bands indicate intact small RNAs. The gels were photographed using UVIsoft image acquisition and analysis software.
